# How to design information strategies on e-mental health services for prospective healthcare providers

**DOI:** 10.1093/eurpub/ckac131.177

**Published:** 2022-10-25

**Authors:** P Braun, A-K Schwientek, A Loerbroks, J Apolinário-Hagen

**Affiliations:** 1 Medical Faculty, Heinrich Heine University Dusseldorf, Dusseldorf, Germany; 2 Department of Psychiatry and Psychotherapy, Technical University of Munich, Munich, Germany

## Abstract

**Background:**

Among prospective healthcare providers (HCPs) such as medical students, mental health problems are prevalent, but they seem reluctant to seek help. Barriers include fear of stigmatization or limited resources of counseling centers. Electronic mental health services (eMHSs) seem to be promising low-threshold and evidence-based tools for increasing treatment availability. However, uptake rates remain low. Reasons include skepticism and lacking awareness, which can be addressed through acceptance-facilitating interventions (AFIs) such as multi-attributes information strategies. To date little is known about how to design AFIs to meet prospect HCPs’ information needs and preferences.

**Methods:**

Between August 2021 and June 2022, n = 21 semi-structured online interviews and n = 3 co-design workshops were conducted with medical and psychology students across Germany to define attributes and levels of information strategies. Interviews were recorded, transcribed and content-analyzed using MAXQDA.

**Results:**

Most students reported having little knowledge about eMHSs but would have liked to be informed at the beginning of their studies or as an integral part of their study program. We identified 5 attributes that information strategies should consist of: information source (e.g., student counseling center), information path (e.g., flyer), timing (e.g., during freshman week), recommendation (e.g., from HCPs), and quality criterion (e.g., evidence-base). Attributes included 4 to 6 levels. Concerning design preferences, students favored green or blue as colors, and short texts with images.

**Conclusions:**

For a comprehensive dissemination of eMHSs into the healthcare system, prospective HCPs need to be educated on eMHSs. This study gives first insight into how AFIs should be designed to inform prospective HCPs. Future research should focus on systematic variations of AFIs’ attributes and their levels mimicking real-world decision scenarios through discrete choice experiments.

**Key messages:**

• Through tailored AFIs, prospective HCPs can be informed about eMHSs for both personal and professional needs.

• Our results give student counseling centers with limited resources clear guidelines on how to inform prospective HCPs on low-threshold eMHSs.

